# President’s Address1Read before the Thirty-fifth Annual Meeting of the United States Veterinary Medica Association, Omaha, Nebraska, September 6, 1898.

**Published:** 1898-09

**Authors:** D. E. Salmon

**Affiliations:** Chief of Bureau of Animal Industry, Department of Agriculture, Washington, D.C.


THE JOURNAL

OF

COMPARATIVE MEDICINE AND VETERINARY ARCHIVES.

VOL. XIX. *SEPTEMBER, 1898.* NO. 9.

### PRESIDENT'S ADDRESS.[1]

BY D. E. SALMON, D.V.M.,

CHIEF OF BUREAU OF ANIMAL INDUSTRY, DEPARTMENT OF AGRICULTURE, WASHINGTON, D.C.

THE welcome that has been extended to this Association by the great city of Omaha, and the prospects for a large attendance of members and other representative veterinarians, give promise that this thirty-fifth annual meeting will be one of the important historic points in the life of our organization. Within the remembrance of some who are now present the veterinary profession has developed from an apparently insignificant beginning in a few of the Eastern cities until now its representatives are found in all sections of the country. There could be no better demonstration of this fact than the gathering here on the banks of the Missouri River of the many gentlemen from the East and from the West, from the North and from the South, who will contribute to the interest and success of this meeting.

As the Executive of this Association, I congratulate you upon the knowledge which we have just acquired, that we can meet here in what many of us have considered a far Western city, and not only receive a cordial greeting, but find ourselves surrounded by sympathetic members of our own profession. And I congratulate the citizens of this great State that there are located here so many competent veterinarians whose lifework it is not only to prevent and cure the maladies of the domesticated animals, but to guard the health of the people from the many diseases which they may acquire by contact with such animals or by consuming as food the various kinds of animal products. The veterinarian has evidently made a place for himself on these fertile prairies, where horses,

[1] Read before the Thirty-fifth Annual Meeting of the United States Veterinary Medica Association, Omaha, Nebraska, September 6, 1898.

cattle, sheep, and swine multiply in such profusion and develop in such perfection; but he has just begun his work, and the more his plans develop, and the better his efforts and aims are understood, the more will he be esteemed and appreciated. In the closing years of this century, which has been so wonderful in scientific progress and achievement, there is nothing more remarkable than the influence which the study of animal diseases has had upon the advancement of human medicine and the resources which the investigators of this subject have laid at the feet of suffering humanity. The elucidation of the nature of contagion; the establishment of scientific disinfection; the development of aseptic surgery; the introduction of bacterial products, vaccines, animal extracts, and antitoxins for the treatment of various diseases are well-known examples.

### THE MICROBES OF CONTAGIOUS PLEURO-PNEUMONIA.

The study of the contagious pleuro-pneumonia of cattle has during the past year revealed a realm of life beyond the reach of the most powerful microscope. For years pathologists have searched for the microbe of this disease without success, and now we learn that their failure was largely due to the fact that this microbe is so extremely small that even the perfect microscopes of the present day are not sufficient to enable the observer to make out its form and dimensions. The ingenious methods of investigation which were adopted in these researches appear to be free from flaws, and we must, therefore, accept the fact that there are living organisms far more minute than have heretofore been recognized, and that, indeed, there is a world of life that the microscope is powerless to reveal, just as we have long known of a world that our unaided vision could not detect.

The bearing of this discovery upon future researches is manifest. There are still numerous communicable diseases of which the active cause has escaped the search of our most able investigators. We have here another clew to these problems, and without doubt they will all finally be resolved by the perseverance and resourcefulness of the modern student.

### VARIATIONS OF TUBERCLE BACILLUS.

The discovery by Dubard of tuberculosis in fish has served to broaden our views concerning this most interesting and destructive panzoötic disease. From a study of the bacillus of mammalian tuberculosis we learned that this microbe requires for its multiplication a temperature between 86° and 104° F., and we concluded from this fact that this germ is an obligatory parasite, unable to multiply outside of the animal body except under special conditions furnished in the laboratory. Later it was discovered that in the tuberculosis of birds, or avian tuberculosis, the bacillus had undergone a remarkable physiological modification, and that it is able to grow all the way from 77° to 113° F.—that is, instead of being confined to a temperature range of 18° F., as is the case with the mammalian bacillus, it has in the avian variety acquired the power to multiply through a temperature range of 36° F. The doubling of the temperature range and the ability to multiply at a point nine degrees lower on the scale have an important signification; for, whereas a continued temperature of 86° F. is difficult to realize in nature, 77° F. for days and nights in succession are not infrequent in many parts of the country. Already the question was suggested as to whether it is not possible for the bacillus tuberculosis to live and multiply in nature as a saprophyte.

Dubard's discovery of tuberculous carp, the bacillus from which is able to grow from 50° to 96.6° F., is still more astonishing, and opens a field of possibilities so extensive that it is safer to wait for the positive results of investigations than to speculate as to what may or may not be true. The facts already established are, however, most important. A bacillus which can vegetate at 50° F. can live as a saprophyte without difficulty, if it finds a proper food supply.

The first question that suggests itself is as to the identity of these bacilli, which are so frequent in their physiological requirements. Are the mammalian bacilli, the avian bacilli, and the piscine bacilli but varieties of the same species, convertible from one to the other, or are they specifically distinct? The researches which have already been made appear to warrant the conclusion that the avian and piscine bacilli may be given all the characteristics of the mammalian form by growing them for a sufficient time under proper conditions. Indeed, Dubard is of the opinion that the carp were infected by throwing into the stream in which they lived the excreta and sputa of a human patient affected with pulmonary and intestinal tuberculosis.

Wide, therefore, as is the gulf which separates the cold-blooded carp from the mammalia, or the latter from the hyperthermic birds; remarkable as are the morphological and physiological differences shown by the bacilli from these different sources, we are forced to the conclusion that these differences are superficial, that they vary with the conditions of environment, and that the tuberculosis of the fish, the mammal, and the bird is one and the same disease.

Accepting this conclusion, that the mammalian bacillus may under certain conditions infect fish and be so modified that it has the vigor to grow at a temperature of 36° F. lower than before, and that, on the other hand, it may infect birds and be so modified as to grow at a temperature 9° F. higher than before, should we not be conservative in adopting the views recently promulgated to the effect that the bovine and human bacilli are different varieties and that the human bacilli are incapable of affecting cattle? This question is one of great importance to the sanitarian, and will no doubt receive your most careful consideration.

### RECENT PROGRESS IN THE CONTROL OF ANIMAL DISEASES.

Much has recently been accomplished in improving and adding to the methods available for the control of a number of our worst plagues. It is not many years since Texas fever was one of the most dreaded diseases, both on account of its destructiveness and of the mystery connected with its origin and dissemination. At last this mystery has been cleared away, and we are to-day in a position to formulate more efficacious regulations for preventing this disease than is possible in connection with most others. By regulating the traffic in cattle from the infected district during the warm season of the year; by allowing them to be moved by rail only and for immediate slaughter during that season; by segregating such cattle and disinfecting the cars in which they are transported, the losses from this disease in the Northern States have been reduced to an insignificant amount.

There, however, remained other problems pressing for solution. In the vast district in which this contagion is enzoötic, comprising practically the entire area of six great States and one Territory, more than half the area of four other States, and important sections of two additional States and one Territory—in this extensive district vast numbers of young cattle are reared to be sold for grazing. It is very difficult, as you will understand, to confine the shipment of these animals to the short period of two months in the winter season. The magnitude of the interests involved is a continual menace to the quarantine regulations. Fortunately, we can now see our way to disinfecting these cattle so that they can be safely shipped anywhere, at all seasons of the year, without conveying the contagion to other animals.

Although these Southern cattle carry the microbe of the disease in their blood for months and years after they leave the infected district, nature has beneficently provided that under ordinary conditions it can only cause disease when transferred to other animals by a single species of external parasite—the Southern cattle-tick, *Boophilus bovis.* With this fact demonstrated, it only remained to discover a practical method of destroying these ticks upon the Southern cattle at the time they passed out of the infected district. This, however, was much easier to propose than to accomplish. Many preparations have been suggested, and it was reported that one of these when tried killed the ticks immediately and the cattle in fifteen minutes. Other mixtures had no effect upon either the cattle or the ticks. It may now be said, however, that in extra dynamo-oil and sulphur we have a dip which kills the ticks with so little effect upon the animals that it can neither be objected to on the ground of financial loss nor cruelty. There may be and doubtless will be further improvements made in the composition of the dipping-mixture used for this purpose, but the point to be emphasized is that we *already* have a dip which may now be successfully used for this purpose.

There is but one other practical problem connected with the prevention of this disease, and that relates to the immunization of cattle that are taken into the infected district. You all know that the cattle of that district are immune, otherwise they would contract the disease and die. Doubtless they obtain their immunity by undergoing a mild attack of the disease while they are young, and if so, why should we not follow the way pointed out by nature, and artificially infect young animals that are destined for the Texas-fever district? This has been successfully done, and it has been shown that the artificially immunized animals were able to resist the disease when taken to the infected section of the country. The principle is thoroughly demonstrated, and it only remains to work out the details of the method by determining the variations which are required acording to the age and breed of the animals and season of the year. We may, therefore, claim with complete justice that the veterinary profession of the United States has not only explained the mysteries of Texas fever, but that it also offers adequate means for the prevention of this disease.

The infectious diseases of swine have long caused such enormous losses that the swinegrowers have been discouraged and many of them financially ruined, while even the Federal Government has been greatly concerned on account of the destruction of property and the menace to an important item of the food-supply and of the export trade. Veterinary science has had much to contend with before it could offer a practical and efficient solution of the problem of preventing these losses. It was necessary to consider the vast number of animals liable to the disease and the great extent of territory over which they are distributed; also, the relatively small value of each individual, and the fact that the losses are caused by two distinct diseases, each of which requires its own specific treatment, while the symptoms are so obscure that it is difficult in the field to distinguish one from the other.

Hygienic surroundings, isolation, disinfection, medical treatment, inoculation, and vaccination were all tried without satisfactory results. In individual cases benefit was undoubtedly derived from the intelligent application of these measures; but the proportion of failures was too great, the success was too uncertain, and, as is to be expected under such circumstances, no general and systematic efforts were made.

Last year some experiments were conducted with the stamping-out system—that is, by killing diseased and infected animals with a view to arresting the multiplication of the contagion. It was shown by these experiments that it is possible to greatly reduce the losses by this radical method; but it requires a large force of men to find all the infected herds in even a single State; it requires a vast sum of money to compensate for the slaughtered animals; and, worse than all, the enforced slaughter and quarantine develops an opposition fatal to the rigid prosecution of this plan of operations over a large extent of territory.

There remained but one resource to which we could turn with hope in the present condition of science. That is the use of anti-toxin serum. The researches made in this direction have shown that it is possible to produce a serum that will immunize animals to both of these diseases and that will also cure both. This treatment was first tried with small animals, such as rabbits and guinea-pigs, in the laboratory, and being successful there, was tested late last year with herds of infected swine. Of about 250 animals in infected herds, over 75 per cent. were saved, while in herds not treated 85 per cent. died. This year the results with about the same number of animals have been even better, and the prospects are that over 80 per cent. of the animals in infected herds may be saved by this method. Considerable quantities of serum will be used before winter, and we shall soon know definitely what results can be depended upon.

In antitoxic serum we have a most valuable agent for the control of swine-diseases, but it can best be used under professional supervision. The State should regard it as an invaluable addition to its resources for eradicating the disease from our territory. If its application is left to the individual farmer, some will use it, but many more will neglect it; and swine-diseases will continue their ravages with slight abatement. If the State adopts it, and provides for its systematic use wherever the infection appears, and requires the disinfection of stockyards and stockcars, it will not be long before swine can be raised with safety and profit, and the fifty or one hundred millions of dollars which are now annually blotted out by this scourge will go into the pockets of our farmers, increasing the wealth and prosperity of the nation.

### THE ARMY VETERINARIAN.

You will learn from the report of the Chairman of the Committee on Army Legislation that nothing has been accomplished toward the organization of a proper corps of commissioned veterinarians for service in the army. The chairman of the Committee on Military Affairs has been favorable to the necessary legislation, but the War Department has persistently objected, and has prevented the accomplishment of this reform which has been urged for so many years by this Association. Is it not incredible that in this practical and up-to-date country the War Department should insist upon being behind all other civilized nations in its organization and equipment of this branch of the service? Why do those who control that department object to skilled, interested, and responsible officers whose duty it would be to examine and pass upon the millions of dollars' worth of horses that are purchased and upon the other millions of dollars' worth that are condemned as unfit for service? Why do they object to responsible experts who would have the authority to secure proper medicines and instruments for the treatment of sick and disabled animals and to direct humane and intelligent treatment?

I shall not attempt to answer these questions in this address, but as the press and people of the country are asking for information as to why the same department is antiquated and inefficient in some other respects, it is possible that Congress may yet undertake a reorganization on modern lines. When that time comes, let us hope among other practical features there will be given to the army a commissioned verterinary service that will not only insure honesty, economy, and intelligence in the purchase and treatment of animals, but that will give the veterinarian and his family the same prospects of a pension in the case of injury or death that are enjoyed by other classes regularly connected with the military organization.

### CHANGE OF NAME.

You will have an opportunity at this meeting to vote upon a proposition to change the name of this Association from the United States Veterinary Medical Association to the American Veterinary Medical Association. This proposition is in line with the growth and development of this body. There are without doubt some disadvantages connected with an enlargement of the field which we represent, but these will probably be more than counterbalanced by the wider range of our vision and the nearer approach to a cosmopolitan character. The practicability of the plan is indicated by the success of the American Public Health Association, which embraces the United States, Canada, and Mexico; but there are some reasons why we should not act without careful deliberation. I trust the matter will be thoroughly considered before action is taken.

### THE PRESIDENTIAL TERM.

There is, also, a proposition to be voted upon to increase the presidential term from one year, as at present, to two years. I regard this proposition as ill-advised and undesirable. We have had numerous members of this Association who should have been honored with the presidency, but for whom the opportunity has never come. If we double the length of the term we lessen by 50 per cent. the chances of every member to gain this distinction, which should be coveted by all. Again, there are worthy members who would like to be President for one year, but who do not feel that they can give the time required by a term of two years. Why should we change the policy of the Association and insist that no member can be elected unless he serves for two years? Finally, if a President is so efficient in the discharge of his duties that the Association desires to avail of his services for two years, and he is willing to serve for that length of time, it is a very simple matter to re-elect him. These considerations lead me to express the hope that this proposed amendment to the constitution will not carry.

### THE WORK OF THIS ASSOCIATION.

Gentlemen, this Association still has a great work before it. Much of the field of animal diseases in this country has never been explored. Concerning the diseases that have been long known and studied, there is still much to learn. We are in the midst of a great public which is ignorant of the principles of medicine in general, and particularly ignorant as to animal diseases and their influence upon the health and wealth of the nation.

The work of this Association must be principally of an educational character. It should begin with its own members, encourage them to study, to think, and to write. It should particularly encourage original observation and investigation. It should use its influence to keep the veterinary literature of the country—the journals, the text-books, and the official reports—abreast with the times and equal, if not superior, to any that are issued in any other country. It should also be active in educating public sentiment.

The citizens of this country, as a body, can be trusted to do the right thing if they thoroughly understand any question. If they have interfered with our work, and in some instances apparently turned the hands backward on the dial of progress, it is because the educational work had been neglected, or, through a lack of discretion, prejudice, and personal feeling have been aroused. Convince the people of the various municipalities that we are laboring to save their property and to protect their health, and it will be strange, indeed, if we meet with opposition.

The great system of meat-inspection, now happily inaugurated, should be carried to perfection, so that the consumer can buy a piece of meat in any market in the country knowing that it has been inspected and that it did not originate from an animal diseased, injured, or otherwise unfit to furnish wholesome food.

A system of milk-inspection should be developed which will guard against filth, the germs of tuberculosis, typhoid fever, and other communicable diseases, and which will make it possible to drink a glass of milk in our cities without serious misgivings as to its effect upon our health.

The animal plagues which now ravage the land, tuberculosis, hog-cholera, and swine-plague, Texas fever, glanders, sheep-scab, and rabies should be rigidly controlled and eradicated.

More attention should be given to the treatment of the ordinary sporadic diseases, so that the veterinarian shall be prepared at all times to give the animal of his client the best treatment which the present condition of science will permit.

To accomplish all of this programme will require years of work, but it is your legitimate work. Individuals will come and go, but as an Association, the illimitable future is yours. There is no task so great, no achievement so distant, that its prospects need discourage you. Let us labor, then, systematically, with full confidence that in time all proper hopes shall be realized.

